# Target-rate effect in continuous visual search

**DOI:** 10.1186/s41235-022-00392-8

**Published:** 2022-05-07

**Authors:** Louis K. H. Chan, Winnie W. L. Chan

**Affiliations:** 1 Psychology Unit, School of Continuing Education, Hong Kong Baptist University, Shek Mun, Hong Kong; 2 Department of Counselling and Psychology, Hong Kong Shue Yan University, North Point, Hong Kong

**Keywords:** Continuous visual search, Dynamic visual search, Target-prevalence effect, Low-prevalence effect, Vigilance, Surveillance, Signal detection

## Abstract

From infrared body temperature surveillance to lifeguarding, real-life visual search is usually continuous and comes with rare targets. Previous research has examined realistic search tasks involving separate slides (such as baggage screening and radiography), but search tasks that require continuous monitoring have generally received less attention. In this study, we investigated whether continuous visual search would display a target-rate effect similar to the low-prevalence effect (LPE) in regular visual search. We designed a continuous detection task for a target feature (e.g., a green color) among items of continuously and gradually changing features (e.g., other colors). In four experiments, we demonstrated target-rate effects in terms of slower hit response times (RTs) and higher miss rates when targets were rare. Similar to regular search, target-rate effects were also observed for relative frequencies across two target features. Taken together, these results suggest a target-rate effect in continuous visual search, and its behavioral characteristics are generally similar to those of the LPE in regular visual search.

## Significance statement

Visual search is a very common task in laboratories and in the real world. In visual search, the observer scans through a visual scene for a designated target. Previous research has studied this task extensively to understand better the involved attentional and decisional processes. Such understanding can help improve visual search performance that bears real-world significance. However, previous research has mainly focused on searches done on discrete scenes, such that each inspection can be finished within seconds. However, many real-world search tasks, such as body temperature surveillance or lifeguarding, are continuous and long. Since the cognitive challenges and decision strategies can vastly differ between short and long visual searches, it is important to determine whether the previous understanding of visual search is generalizable to the long, continuous variant. In the current study, we found that a low target prevalence is associated with more target misses in continuous visual search, similar to regular visual search. Our findings indicate that the decisional process between the two kinds of search may have a lot of commonalities. Therefore, in prescribing instructions, precautions, or trainings for real-world continuous search, previous knowledge and experience with regular visual search may have high reference value.

## Target-rate effect in continuous visual search

Since the outbreak of the coronavirus pandemic, infrared body temperature surveillance has become very common. Monitoring this kind of display is a visual search task—a task that has been studied extensively to understand visual attention and its real-life significance (for a review, see Wolfe, 2020). Notably, previous research has mainly focused on visual search tasks involving separate, static displays. However, visual search tasks like body temperature surveillance involve continuous and dynamic displays. Real-life tasks of a similar nature are quite common. Lifeguarding and security surveillance are some examples.

*Continuous visual search* task has two main characteristics. First, it involves a search component: searching for a target among multiple distractors. Second, it involves a monitoring component: monitoring for an unknown number of critical events over a period of time with only non-critical events. Previous research has generally covered one but not the other characteristic. For instance, visual search literature has typically studied multi-item searches in discrete trials (e.g., Treisman & Gelade, 1980; Wolfe et al., 1989). In such tasks, participants look for a predefined target (e.g., a red circle) among multiple distractors (e.g., several green circles). The stimulus display is generally static and presented one after the other. In each trial, zero or one target may exist, and the participant has to determine whether a target is present for each trial. Usually, it does not take long to inspect each search display. This laboratory task may approximate real-life tasks, such as baggage screening (e.g., Wolfe et al., 2005) and medical image inspection (e.g., Wolfe, 2016). In both tasks, the searcher (security officer or radiologist) has to search among multiple distractors (other common objects in each bag or other bodily tissues visualized in each image). Moreover, both tasks involve discrete inspections (individual bags, individual medical images).

Meanwhile, vigilance literature has studied continuous monitoring for critical events, but only one object is typically monitored. For example, Mackworth (1948) asked participants to detect an occasional “double tick” on an otherwise normally ticking clock. Each participant has to monitor for the abnormal event for a longer period, but this event can only happen to one object, namely the clock arm. There is no search component in this task. This situation is similar to fishing using a fishing float. You monitor the float until it dips, and then you pull. It can take a short or long time, but you do not need to search among other floats. Vigilance tasks usually involve temporary targets, and the participant has to respond to them before they disappear. They withhold their response when there is no target.

Notably, continuous visual search has both search and monitoring components, making it a research gap that is yet to be addressed. Furthermore, many continuous visual search tasks are not simply a hybrid of visual search and vigilance tasks but involve more dynamic, ill-defined, and possibly rare target events. Take lifeguarding as an example. The lifeguard has to search for drowning behaviors among many patrons in a several-hour shift. The body movements of patrons are dynamic and ambiguous. The number of drowning events is rare and unknown, and the drowning motion is temporary. These common characteristics of continuous visual search present extra perceptual and decisional challenges to the observer.

Previous research on continuous visual search is rare. In vigilance literature, some studies have looked at vigils that involve search. However, these studies are usually about how vigilance decrement may happen during lengthy sessions of regular searches. For example, Horowitz et al. (2003) studied how sleepiness affected the visual search for spatial configuration and conjunctive targets; Ghylin et al. (2007) studied vigilance decrement in an x-ray inspection task. While these studies examined vigilance performance in visual search, neither task had a continuous and dynamic property. Drury et al. (2007) studied vigilance performance in aircraft turbine blade inspection, a task that had a more continuous nature in the sense that it took a longer time to inspect the turbine blades for cracks, and the inspection session was not separated into short trials. Furthermore, cracks were probably rare as well. These properties are common in continuous visual search. However, airframe cracks are static and permanent. Therefore, the task is different from continuous search tasks with dynamic and temporary targets. Furthermore, it is difficult to compare airframe inspection with mainstream visual search studies because searching for blade cracks usually involves walking around the airframe. In most mainstream studies, the search is performed in front of a monitor display.

In visual search literature, there have been efforts on visual searches with dynamic properties. Kunar and Watson (2011, 2014) investigated whether previous findings on visual searches with relatively simple and static stimuli may be generalized to more dynamic and heterogeneous search elements. In their study, they created a so-called multielement asynchronous dynamic (MAD) search task. Compared with regular visual search tasks, a MAD search uses larger set sizes, with half of the search items moving in random directions and speeds and half of the search items oscillating asynchronously in terms of luminance. The target identity is variable (which could be one of the five vowel letters). This way, the MAD task is dynamic and less predictable. In six experiments, Kunar and Watson (2011) found two main differences between MAD and regular search. First, item motion made the search less efficient in larger set sizes; second, a higher error rate was associated with an uncertain target identity.

Although a MAD search involves dynamic properties, it is a trial-by-trial search task instead of a continuous one. Nevertheless, Kunar and Watson’s (2011, 2014) studies are valuable in showing that some well-known visual search behavior in regular search conditions may not necessarily be generalized to less typical search settings. Since these settings may be common in the real world, with dynamic features and large set sizes as examples, they warrant investigation. Continuous visual search is similar to this regard in the sense that it mimics many aspects of real-world searches that are uncommon in regular visual search and vigilance tasks, including dynamic features and continuous monitoring. Therefore, it is worth studying whether such properties may render any differences in terms of some well-known search behaviors.

In this study, we aim to test whether the low-prevalence effect (LPE; Wolfe et al., 2005) may be replicated in continuous visual search. The LPE is a well-studied line of research that addresses a research gap between laboratory and real-life visual search tasks. For instance, whereas targets typically occur in 50% of trials in the lab, they are usually less prevalent in real-life searches, such as baggage screening or medical image inspection. Wolfe et al. (2005) found that at a low target prevalence, miss rates can be extremely elevated. In many real-life continuous visual search tasks, targets are also rare. For example, neither drowning nor an elevated temperature is a very common event in a lifeguarding or body temperature surveillance session. Therefore, there is a practical significance in investigating how the LPE generalizes from regular to continuous visual search.

Furthermore, an analysis of the similarities and differences between regular and continuous visual search also suggests that it is a topic that is of theoretical importance. In regular visual search, the LPE is most discussed in terms of a change in decision criteria (Wolfe & Van Wert, 2010; Wolfe et al., 2007), perceptual failure (Godwin et al., 2015; Hout et al., 2015), early termination (e.g., Rich et al., 2008; Wolfe et al., 2005), and habitual responses (Fleck & Mitroff, 2007). To briefly summarize each account, first, a low target prevalence may shift the decision criteria toward a conservative direction and lead to more misses when a target is identified. Second, a low target prevalence may reduce the attentional priority of selecting and identifying the target. Third, a low target prevalence may lead to a shorter and less complete search. Fourth, a low target prevalence may lead to a habitual motor response for the target-absent button. A brief analysis of each account shows different applicability to regular and continuous searches. First, continuous visual search should still be subject to decision bias and perceptual failure due to rare targets. However, due to the task’s nature, one cannot terminate the search prematurely in a continuous visual search until the whole search session is completed. Moreover, in a continuous visual search, one typically responds to the presence of a target only, withholding any response when no target is present. This makes it unlikely for habitual responses to cause any LPE in a continuous visual search. Therefore, studying LPE in continuous visual search helps in understanding the locus of LPE in visual search in general.

An aim of the present study is to develop a general paradigm that abstracts the core features of real-life continuous visual searches while keeping them as laboratory tasks that are easy to control and manipulate. This paradigm is designed to possess four features: multiple search items, continuous monitoring, dynamic search features, and temporary targets. Therefore, in these search tasks, all items have a constantly changing feature. Most times, they have a non-target feature. Occasionally, such a feature may transform from a non-target to a target one, and the item becomes a target. The searcher has to search in this dynamic scene for several minutes and make a response when any target is seen. Since a target can appear (and then disappear) at any moment, the search is continuous and not separated into discrete trials. In this study, we use this paradigm to test the LPE in continuous search in four experiments.

In Experiment 1, we first attempt to replicate the basic LPE, or what we call the target-rate effect in a continuous search, where target prevalence is defined by the target occurrence rate in a continuous time domain. The participants searched for a target color (a green color) among an array of color-changing items (non-green). The target color occasionally occurred, stopped changing briefly, and changed back to a non-target color. The rate at which the target color occurred was manipulated. In regular visual search, a lower target prevalence is associated with a higher miss rate and sometimes slower hit response times (RTs) (Wolfe et al., 2005; Wolfe et al., 2007; Wolfe & Van Wert, 2010). Similarly, in vigilance studies, a lower signal probability is associated with a higher miss rate (e.g., Colquhoun, 1961). Therefore, it is reasonable to expect a higher miss rate and a slower hit RT in this experiment. As a preview, our results matched this expectation.

In Experiment 2, we extended the investigation to examine a relative target-rate effect, in which participants searched for two target features presented at different rates. Previous visual search studies (Godwin et al., 2015; Hout et al., 2015; Wolfe et al., 2007) have shown that when targets of high and low prevalence were mixed, a relative prevalence effect would occur, such that the relatively rare target was missed more often than the relatively frequent target. One motivation for studying the relative prevalence effects in previous studies was to control for the general vigilance level, motor error, and premature search termination in the search because these variables should affect the detection of both targets. Therefore, it is of our interest to ascertain whether such an effect would be replicated in continuous visual search. As a preview of our results, we found a relative target-rate effect in terms of a higher miss rate and slower hit RTs for the relatively rare target.

In Experiment 3, we manipulated the search set size and investigated whether the relative target rate would affect the search efficiency. Previous visual search studies have shown that relative target prevalence did not only affect the post-selective perceptual identification of targets but also influenced the speed of perceptual selection, as long as the two targets have dissimilar features (Godwin et al., 2015; Hout et al., 2015). The faster selection was evident by a quicker first fixation on the relatively frequent target, meaning that attention was preferentially guided to it. In this experiment, we are interested in knowing how this finding can be generalized to continuous visual search. We operationalized the speed of perceptual selection in terms of search efficiency (i.e., set-size effect), which reflected the effectiveness of attentional guidance to the target. As a preview of the results, we found that the more frequent target was associated with a more efficient search.

Experiment 4 serves two purposes. First, we measured the search efficiency in an absolute target-rate manipulation. Although relative target prevalence seemed to affect the perceptual selection, previous studies have generally argued against a perceptual selection account for LPE in general (Godwin et al., 2015; Hout et al., 2015). Therefore, we expected no target-rate effect on search efficiency in an absolute design. Our result matched this expectation. More importantly, rare targets were still associated with more misses and slower hit RTs, demonstrating a regular target-rate effect. This confirmed the previous understanding of LPE in visual search.

The second purpose of Experiment 4 was to evaluate a vigilance account for target-rate effects. Although we used relative target-rate manipulation to control for vigilance levels in Experiments 2 and 3 and still found an effect, it does not mean that the vigilance difference between frequent and rare conditions cannot have its own contribution to the effect. Therefore, we used a flash detection paradigm to measure the vigilance levels in high and low target-rate conditions. The results showed that the different target rates were not associated with the different vigilance levels.

## Experiment 1

**Fig. 1:**
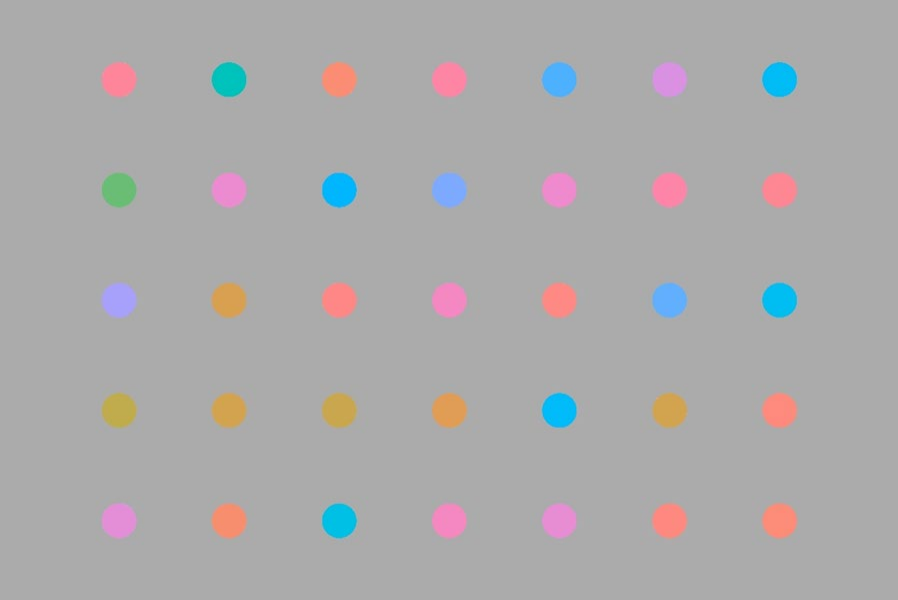
Sample search display in Experiment 1. *Note* In Experiment 1, each search item was a circle filled with a changing color. When an item changed to green (e.g., the left-most item in the second row), it became a target, and the participant had to detect it (online demo available at: https://louiskhchan.github.io/dynamic_search_demo/)

We began our investigation by first establishing a basic target-rate effect in a continuous visual search paradigm, analogous to an LPE in regular visual search. Participants searched for a target color (a medium green color) among an array of color-changing items. At the start, all the items had a non-target color, which changed and stopped from time to time. Occasionally, with its frequency depending on the target rate, one item might change to the target color and stop for 2 s (see Fig. 1 for a sample display). The participants had to push the space bar once they spotted the target before the item changed back to a non-target color again. There were two target-rate conditions, namely frequent and rare, at rates of 60 and 10 items in each 15-min session, respectively. We expected a higher miss rate and slower hit RTs for the rare target condition.

### Method

#### Participants

A total of 44 undergraduate students were involved in the study, with 38 participating as part of a class project; the rest participated with monetary compensation. Most of them came from Hong Kong Baptist University, and a few were from other local universities in Hong Kong.

#### Apparatus and stimuli

The experiment was programmed using JavaScript and conducted online. Graphics were presented using HTML5 Canvas. The participants used their own computers to run the experiment. Stimulus dimensions were set relative to the screen height of the participants’ computers. For presentation purposes, in this report, we used a fixed conversion from each percentage of user screen height to 0.2° visual angle when reporting stimulus dimensions. This conversion would approximate a 60-cm viewing distance with a 17″ display or a 50-cm viewing distance with a 14″ display.

The search display consisted of 35 color disks arranged on a 7 × 5 grid 18.2° wide and 13° tall. Each color disk had a diameter of 0.8°. Stimulus colors were specified in the LCH color space and converted into RGB values using the chroma.js library. Briefly, the LCH color space is based on the CIELAB color space to provide approximate perceptual uniformity for color distances, but it uses a polar coordinate so that colors can be specified in a similar fashion to the HSL model. Here, L represents perceptual lightness, C refers to chroma (which has a similar meaning to the saturation of color), and H represents the hue angle. The possible value of the hue angle varies from 0° to 360°. In Experiment 1, the background color was lch(70,0,0), which corresponds to a color with 70% luminance and zero saturation (i.e., a light gray color). All stimulus disks had colors of lch(70,50,*H*), in which *H°* was the current hue of the disk. In our experiment, we defined a 145° hue, a green hue, as the target. To avoid ambiguity in distinguishing the target color, no search items had a color nearer than 45° from the target hue when they were not targets. As such, at the beginning of each session, the beginning hue of each disk was a random hue between 0° and 100° or 190° and 360°. To recap, 145° is the target hue, and 100°–190° is a reserved hue zone. Outside of the reserved hue zone is the non-target hue zone. As the experiment progresses, the color of each disk gradually changes toward another random hue in the non-target zone at a rate of 100°/second. They would never go across the reserved hue zone. Once a disk arrives at a color stop, its color would stop changing for 2 s. Then, it begins another journey to yet another random non-target hue. Occasionally, a disk is chosen as a target. In this case, its next color stop would have the target hue. It would gradually change to the target hue, stop for 2 s, and then change to a random non-target hue afterward.

#### Procedure

The experiment link was distributed to the participants in class. Before the experiment began, instructions were shown on the experiment website, together with a half-minute demo session with immediate feedback to ensure understanding of the experiment. The demo session was repeated until the detection performance was satisfactory. The experiment contained two 15-min sessions; 60 and 10 targets were presented in the frequent and rare target sessions, respectively. The session order was counterbalanced across the participants.

Each session of either target rate had a dynamic search display, as described earlier in the *Apparatus and Stimuli* section. Each session was split into equal time intervals according to the prescribed number of targets, and one target was assigned within each interval. Within each interval, a random time point was selected. As the experiment progressed to that time point, the next color stop of a randomly selected item would be set as the target color and become a target.

When the participants see any of the target colors, they must press the space bar immediately. Response time (RT) was defined as the time interval between the target just arriving at the target hue (145°) and the corresponding key press. Since no non-target items could ever enter the reserved hue zone (100°–190°; see *Apparatus and Stimuli* above), theoretically, one could start detecting a target even when it is still approaching. Therefore, we registered any response made when the target color was within the reserved hue zone as a correct hit response. Since each target needs to enter the reserved hue zone, stop at the target hue for 2 s, and then leave the reserved hue zone, the possible hit RT range was from − 450 to 2450 ms. If a response was not made within this interval, it was counted as a miss. If a response was made when no item was a target, it was counted as a false alarm.

### Results

**Fig. 2:**
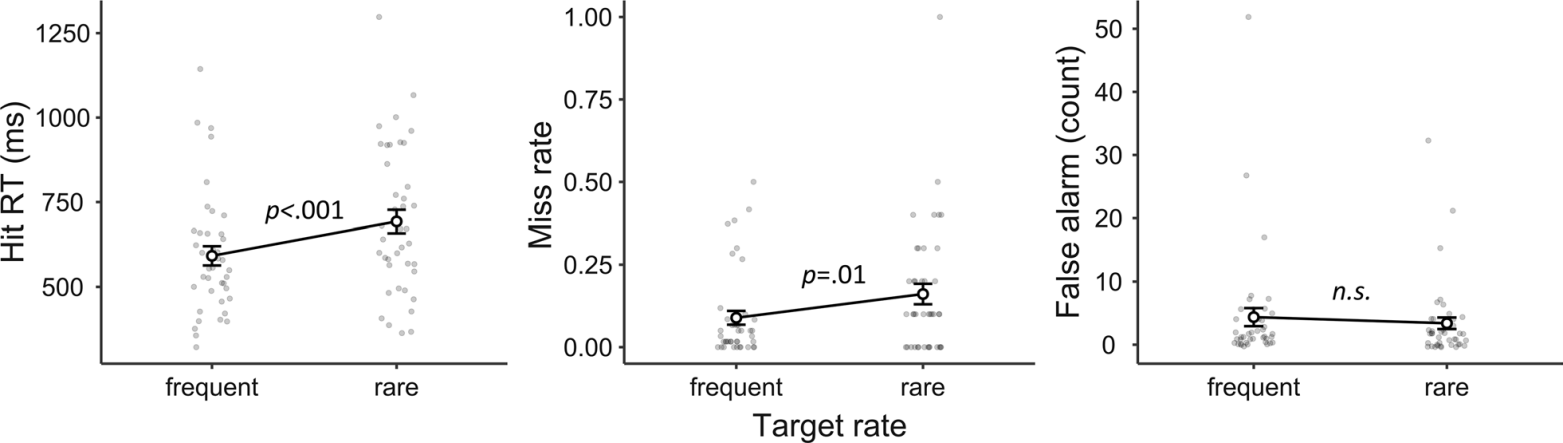
Experiment 1 Results. *Note* In Experiment 1, target-rate effects were observed on hit RTs and miss rates, but not false alarms. Error bars represent ± 1 standard error of the mean

We analyzed our data in terms of hit RTs, miss rates, and false alarm counts. Data from three participants were not analyzed due to excessive false alarms (> 200), and data from one additional participant were not included in the hit RT analysis due to a 100% miss rate in one condition. For each dependent variable, we conducted a two-way mixed-design analysis of variance (ANOVA) to compare the two target-rate conditions (frequent vs. rare), with session order entered as a between-subject variable to check for any order effect. To evaluate any null effects, we also conducted a Bayesian ANOVA of the same design alongside each respective ANOVA. We used the Bayesian methods module (jsq 1.0.2) in Jamovi 2.2 (The Jamovi Project, 2022) to conduct the Bayesian ANOVA. For each effect, we compared the matched models including or excluding it and reported a Bayes factor (${\text{BF}}_{{\rm inclusion}}$) that indicates the inclination toward including such an effect (Mathôt, 2017). The descriptive results are shown in Fig. 2.

#### Hit RTs

The ANOVA and Bayesian ANOVA results showed significantly and strongly slower hit RTs in the rare condition than in the frequent condition ($F\left({1,38}\right)=13.54, p<.001, {\eta }_{p}^{2}=.26; {\text{BF}}_{{\rm inclusion}}=40.16$). No effects involving session order were significant (all $p>.3$).

#### Miss rate

The miss rate was calculated as the percentage of missed targets. The ANOVA and Bayesian ANOVA results showed a significantly and substantially higher miss rate in the rare condition than in the frequent condition ($F\left({1,39}\right)=7.16, p=.01, {\eta }_{p}^{2}=.16; {\text{BF}}_{{\rm inclusion}}=4.62).$ No effects involving session order were significant (all $p>.8$).

As one participant produced a 100% miss rate in the rare session, it is reasonable to wonder whether the significant target-rate effect critically relied on these data. Therefore, we excluded this result and conducted the analysis again. The ANOVA and Bayesian ANOVA results still showed a significantly and substantially higher miss rate in the rare condition than in the frequent condition ($F\left({1,38}\right)=6.58, p=.01, {\eta }_{p}^{2}=.15;{\text{BF}}_{{\rm inclusion}}=3.52).$

#### False alarms

The number of false alarms was analyzed because there was no equivalence of the “number of target-absent trials” in continuous visual search to serve as the base of a ratio measure. ANOVA revealed no significant effect of the target rate ($F\left({1,39}\right)=.28, p=.60, {\eta }_{p}^{2}=.00$). Bayesian ANOVA showed a substantial inclination toward the lack of a target-rate effect (${\text{BF}}_{{\rm inclusion}}=0.27$). No effects involving session order were significant (all $p>.05$).

### Discussion

The continuous visual search task demonstrated the expected target-rate effects on both hit RTs and miss rates. For instance, a higher miss rate and slower hit RTs were observed when the targets were rare. There was no significant difference in terms of the number of false alarms.

Although these target-rate effects are expected, they are open to several possible explanations. First, it could be that less exposure to targets in rare conditions resulted in a poorer perceptual representation of the target. Thus, detection performance was poorer. We believe this possibility is not very likely because it would presuppose a smaller target-rate effect in our frequent-session-first participants in Experiment 1. When these participants encountered their rare sessions, they should have had enough exposure to the target feature already, leading to a better performance in the rare condition and a smaller target-rate effect as a result compared with the rare-session-first participants. However, we did not observe any interaction between session order and target-rate effects in either hit RTs or miss rate.

Second, it could be that the more frequent targets have led to habitual present responses, leading to faster hit RTs and fewer misses. However, this account should lead to more false alarms in the frequent condition, which was not observed. Furthermore, the search targets in our experiment were not very frequent even in the frequent condition and, thus, were unlikely to drive habitual responses.

Third, it could be that we are less vigilant with rare targets overall. In other words, less frequent targets may engage our attention less and produce more attentional lapses, leading to more misses and slower responses. Experiment 2 tested this possibility.

## Experiment 2

Experiment 2 addresses a general vigilance account. Previous vigilance studies have shown that vigilance decrement could occur in a simple monitoring task in as short as 10 min (Molloy & Parasuraman, 1996). In addition, Thomson et al. (2015) showed that the task-relevant complexity could alleviate vigilance decrements by engaging participants in the task. When the target rate is manipulated across sessions, it is possible that participants may not be attentionally engaged as much in a rare target session compared with a frequent target session. As such, a target-rate effect on search performance may result from different vigilance levels across target-rate sessions.

Previous research in visual search has manipulated relative target prevalence to control for vigilance levels across prevalence conditions. By mixing two target types and manipulating their relative prevalence, their overall prevalence can be kept constant across the manipulation. Hence, the participants must have the same vigilance levels regardless of whether the frequent or rare target is being detected. Previous studies (e.g., Godwin et al., 2015; Hout et al., 2015) have generally found that relative prevalence still leads to LPEs, suggesting that vigilance levels cannot fully explain LPEs. Other reasons, such as perceptual and decisional limitations when detecting rare targets, must be taken into account. Relative target prevalence studies are also practically important because they indicate whether mixing non-critical targets among rare critical targets is a good way to “cure” the LPE (e.g., Wolfe et al., 2007).

**Fig. 3:**
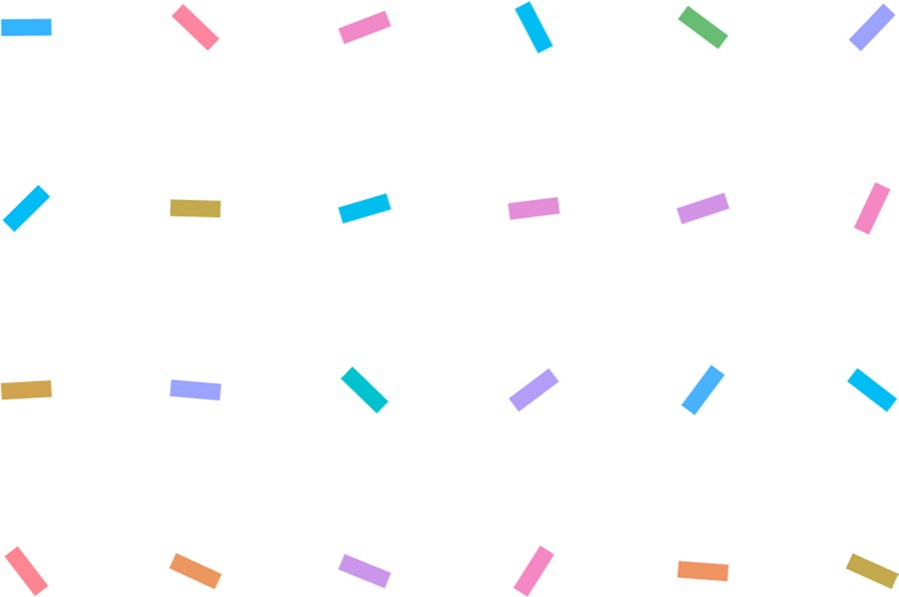
Sample search display in Experiment 2. *Note* In Experiment 2, each search item has a changing color and rotating orientation, and any green or vertical targets must be detected. In this image, the second right-most item on the top row was a green color target (online demo available at: https://louiskhchan.github.io/dynamic_search_demo/)

In the same vein, we attempted to address a general vigilance account by mixing two target types and manipulating their relative rates in Experiment 2. We asked the participants to search for both a target color (green) and a target orientation (vertical) among color-changing and rotating bars (Fig. 3). To manipulate the relative rate of each target, there were two relative-rate conditions, namely the more-color-target condition and the more-orientation-target condition, both of which were manipulated across sessions. As such, if there was a relative target-rate effect in this continuous search task, we should expect slower hit RTs and higher miss rates for the relatively rare target in each session (i.e., color target in the more-orientation-target condition and orientation target in the more-color-target condition). In other words, we expected interactions between the target types and relative-rate conditions.

### Methods

#### Participants

Thirty undergraduate students who were mainly from Hong Kong Baptist University and the rest from other local universities in Hong Kong took part in the experiment.

#### Apparatus and stimuli

Similar to Experiment 1, the second experiment was programmed and conducted online, with the following modifications. The search display contained 24 color bars arranged on a 6 × 4 grid 18.2° wide and 13° tall. The background color was changed to white to enhance visibility. The color bars were 0.3° wide and 0.9° tall. For the search items, their colors were assigned and varied in the same way as in Experiment 1; their orientations were also assigned and varied similarly, and this was done independently from the colors. The target color value and zone were the same as in Experiment 1. The target orientation was vertical (90°), and the target orientation zone was 67.5°–112.5°, spanning a 45° range around the target orientation. The items rotated at a rate of 50°/second when they were rotating.

#### Procedure

The procedure was based on Experiment 1, with the following changes. The experiment link was sent to the participants via WhatsApp after they received our invitation and agreed to participate. Before the experiment began, online instructions were given, followed by three half-minute demo sessions. The three demo sessions enabled the participants to practice detecting color targets, orientation targets, and both targets mixed, respectively. Each demo session was repeated until the detection performance was satisfactory. Then, the experiment proper began. The experiment was composed of two 15-min sessions: One session had more color targets and the other had more orientation targets. Session order was counterbalanced across the participants. In each session, the more frequent target feature occurred 60 times and the less frequent target feature occurred 10 times in randomly shuffled orders, as described below.

Each test session began with a 6 × 4 array of color bars varying continuously in both colors and orientations, as described in *Apparatus and Stimuli* section. To recap, all items had non-target colors and orientations at the beginning, and then, the color and orientation of each item changed continuously and stopped occasionally. At any moment, the color and orientation of each item were heading toward a next stop value at a constant speed. Once a feature arrived at its stop value, it would stop changing for 2 s, and then it would update its next stop value to another random, non-target feature value. This process was concurrent and independent for the item colors and orientations. Each session was split into 70 equal time intervals. The color and orientation targets were randomly distributed into these time intervals, such that there was one target within each interval. Note that no two targets, whether color or orientation, would occur in the same time interval to avoid response ambiguity. Similar to Experiment 1, at a random time point selected from within each interval, a random item would be selected. The next color or orientation stop of the selected item would be set to the target color or orientation, depending on the target type assigned to that interval. For the non-target feature of that item, it would continue to vary as usual. For example, if in a particular interval a color target was assigned, a random item would be selected with its next color stop set to medium green, and its orientation would still vary within the non-target zone.

In the experiment, the participants had to immediately press the “z” key once they saw a color target and press the “/” key once they saw an orientation target. Similar to Experiment 1, the RT was defined as the duration between the target feature arrives (145° hue or 90° orientation) and the participant responses. A correct hit response would be registered as long as a response was made when the target feature was within the target zone (100°–190° hue or 67.5°–112.5° orientation). The definitions of miss and false alarm were the same as those in Experiment 1.

### Results

**Fig. 4:**
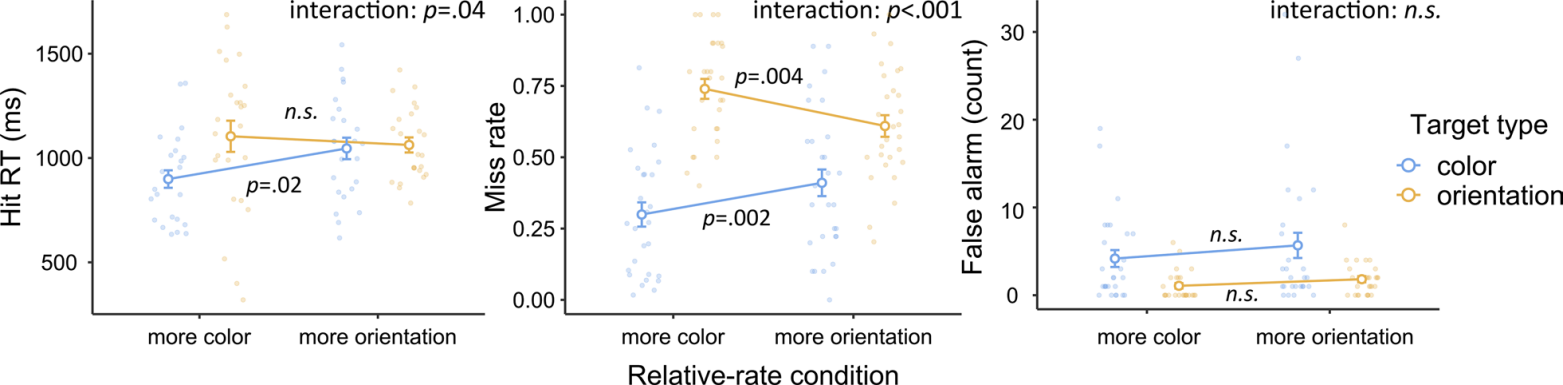
Experiment 2 results. *Note* In Experiment 2, relative target-rate effects occurred mainly in terms of miss rates, such that the relative-rate conditions significantly modulated the performance of detecting each type of target. The same target type was missed more when it was relatively rare. There was a similar relative target-rate effect in terms of hit RTs, but the effect was more visible with color targets. Error bars represent ± 1 standard error of the mean

Data were examined in terms of hit RTs, miss rates, and false alarm counts. Data from two participants were not analyzed due to excessive false alarms (> 50); data from four additional participants were not included in the hit RT analysis due to a 100% miss rate in one or more conditions. For each dependent variable, a three-way mixed-design ANOVA was conducted to study the relationship between the relative-rate condition (more-color-target or more-orientation-target session) and the target type (color or orientation). Session order was entered as between-subject variable to check for any order effect. Similar to the last experiment, the corresponding Bayesian ANOVA was also conducted to evaluate the null effects. The descriptive results are shown in Fig. 4.

#### Hit RTs

We wanted to establish whether performance was affected by whether or not the relative-rate condition matched the target type. In other words, we determined whether the color/orientation targets were detected more quickly in the more-color-target/more-orientation-target conditions, respectively. Consistent with our expectation, ANOVA showed that the relative-rate condition × target type interaction was significant ($F\left({1,22}\right)=4.78, p=.04, {\eta }_{p}^{2}=.18)$. However, the Bayesian ANOVA favored including this effect only non-substantially (${\text{BF}}_{{\rm inclusion}}=1.32)$. Focusing on each target type specifically, only the color targets showed a significant relative target-rate effect ($t\left(22\right)=2.50, p=.02$), in which hit RTs were faster in the more-color-target condition. However, the orientation targets did not show a relative target-rate effect ($t=0.57, p=.58$). There were no other effects observed by ANOVA: relative-rate condition, $F\left({1,22}\right)=1.08, p=.31, {\eta }_{p}^{2}=.05$, target type, $F\left({1,22}\right)=3.63, p=.07, {\eta }_{p}^{2}=.14$. There were no session order-related effects either (all $p>.1$).

#### Miss rate

The ANOVA and its Bayesian counterpart revealed a significant and decisive interaction between the relative-rate condition and the target type [$F\left({1,26}\right)=22.38, p<.001, {\eta }_{p}^{2}=.46; {\text{BF}}_{{\rm inclusion}}=136.52$]. As shown in Fig. 4, this interaction effect indicated that the miss rate of each target type was higher when such target type was relatively rare, consistent with our expectation. Focusing on each target type, color targets were missed more in the more-orientation-target condition [$t\left(26\right)=3.41, p=.002$] and orientation targets were missed more in the more-color-target condition [$t\left(26\right)=3.12, p=.004$]. These results were consistent with our expectations.

The ANOVA and its Bayesian counterpart also showed significantly and decisively more misses for orientation targets in general [$F\left({1,26}\right)=70.49, p<.001, {\eta }_{p}^{2}=.73;{\text{BF}}_{{\rm inclusion}}>1000$]. This may reflect that our orientation stimuli were less visually salient than our color stimuli, consistent with our participants’ reports. There was no overall difference between relative-rate conditions [$F\left({1,26}\right)=0.12, p=.73, {\eta }_{p}^{2}=.00; {\text{BF}}_{{\rm inclusion}}=0.20$]. There were no session order-related effects, (all $p>.2$).

#### False alarms

There was no relative target-rate effect on false alarms. The interaction between relative-rate condition and target type was not significant, and Bayesian analysis substantially favored the lack of this effect ($F\left({1,26}\right)=0.28, p=.60, {\eta }_{p}^{2}=.01; {\text{BF}}_{{\rm inclusion}}=0.28$). There were significantly and decisively more false alarms for color targets than for orientation targets [$F\left({1,26}\right)=11.88, p=.002, {\eta }_{p}^{2}=.31; {\text{BF}}_{{\rm inclusion}}=362.29$].

There was a significant but non-substantial interaction between relative-rate condition and session order ($F\left({1,26}\right)=4.26, p=.05, {\eta }_{p}^{2}=.14; {\text{BF}}_{{\rm inclusion}}=0.87$). This interaction was driven by slightly more false alarms in the more-orientation-target condition in the orientation-first participants ($M=5.18$) compared with other conditions ($M=2.32-2.64$). We think this effect was not important for our purpose. There were no other significant effects on false alarms: relative-rate condition, $F\left({1,26}\right)=2.58, p=.12, {\eta }_{p}^{2}=.09; B{F}_{inclusion}=0.50$; for all other session order-related effects, $p>.05$.

### Discussion

Similar to previous findings on relative prevalence effects (e.g., Godwin et al., 2015; Hout et al., 2015), we found a significant interaction between the relative-rate condition (more-color-target versus more-orientation-target sessions) and the target type (color or orientation targets) on hit RTs and miss rate data. Higher miss rates were observed for the target type that was presented relatively rarely. Slower hit RTs were observed for the color targets when they were relatively rare. Overall, we observed a relative target-rate effect on miss rates and hit RTs in this experiment.

While the relative target-rate effect was strong and robust on miss rates, it was less prominent (but still observable) in terms of hit RTs. Only the relative target-rate effect with color but not orientation targets reached statistical significance. This was possibly due to the high miss rates associated with our less salient orientation targets, such that they did not leave a sufficient hit count to maintain a statistically stable estimation of mean RTs. This was not unusual since the corresponding effect in Godwin et al.’s (2015) study was also less robust. Similar to Experiment 1, there was no relative target-rate effect on false alarms.

Since a relative target-rate effect was observed when the overall target rate was held constant, it was not explained by a general vigilance account. The current results also indicated that adding non-critical targets was not a good way to “cure” the target-rate effects in continuous visual search.

## Experiment 3

In the previous experiment, we observed a relative target-rate effect on hit RTs and miss rate. In Experiment 3, we examined whether this effect can be generalized to search efficiency. In visual search, search efficiency measures the effectiveness of attentional guidance (Wolfe, 1998). If an observer knows what visual features to look for, such knowledge can often prioritize the allocation of attention to locations that contain such features, leading to high search efficiency (i.e., a small set-size effect; Wolfe, 1994, 1998) and a quick first fixation to the target (Hout & Goldinger, 2015). Notably, it is interesting to determine whether target rate influences search performance via the effectiveness of attentional guidance.

Previous research on relative LPE has generally found better attentional guidance with a higher target prevalence. When two targets have dissimilar features, both Godwin et al. (2015) and Hout et al. (2015) found quicker first fixations to target with higher target prevalence. This result indicates that attentional guidance is sensitive to target prevalence, such that it is biased toward the more frequent target, leading to a more efficient search. However, when the two targets have similar features, no relative LPE was observed in terms of quicker first fixations with the more frequent target. This is because the perceptual code to support effective attentional guidance is relatively coarse (Anderson, 2014; Nagy & Sanchez, 1990). Thus, one cannot attentionally prioritize one target feature over the other effectively. Nevertheless, relative LPE was still observed in terms of post-fixation identification time, showing that relative target prevalence influenced post-selective perceptual identification performance.

**Fig. 5:**
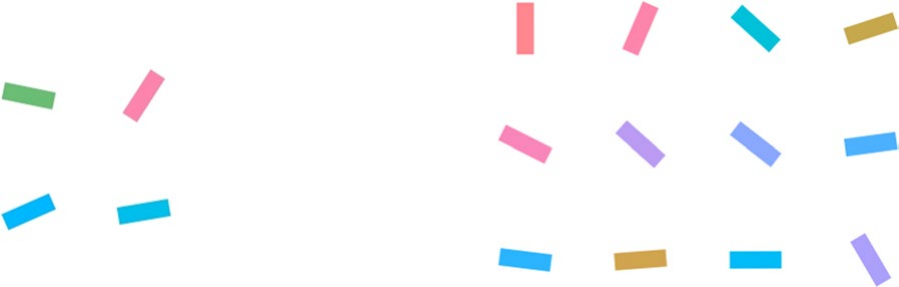
Sample search displays in Experiments 3 and 4. *Note* In Experiment 3, each search item has a changing color and rotating orientation, and any green or vertical targets must be detected. The left panel showed a small set-size display with a color target (the top left item), and the right panel showed a large set-size display with an orientation target (the top left item). In Experiment 4, only vertical targets must be detected. No color targets were shown, and the color variation was irrelevant to the task (online demo available at: https://louiskhchan.github.io/dynamic_search_demo/)

In the last two experiments, we were not able to tell whether the target rate influenced the effectiveness of attentional guidance because we only measured the absolute hit RTs and miss rates. Therefore, in Experiment 3, we also measured search efficiency by manipulating the relative target rate and set size (4 vs. 12) in continuous visual search. Figure 5 shows an illustration of the manipulation. We chose smaller set sizes in this experiment to make the task easier and avoid a high miss rate. In the last experiment, a high miss rate associated with orientation targets appeared to have affected the statistical stability of the hit RT measurement. The experiment was otherwise similar to Experiment 2.

### Method

#### Participants

A total of 26 undergraduate students from Hong Kong Baptist University and other local universities took part in the experiment with monetary compensation.

#### Apparatus and stimuli

The apparatus and stimuli used were the same as those in Experiment 2, except for the stimulus arrangements. In Experiment 3, there were two set sizes: 4 and 12. When the set size was 4, the color bars were arranged on a 2 × 2 grid 4° wide and 4° tall. When the set size was 12, they were arranged on a 4 × 3 grid 8° wide and 6° tall.

#### Procedure

The procedure was the same as that in Experiment 2, except for the ordering of sessions. In this experiment, there were four sessions in total. There were two sessions for each relative-rate condition, one for each set size. Half of the participants started with two consecutive more-color-target sessions, followed by two consecutive more-orientation-target sessions. The other half was the reverse. Among each half of the participants, half of them (i.e., a quarter of all) started with a small set-size session, followed by a large set-size session. The other half was the reverse. Different from Experiment 2, each session lasted for 10 min.

### Results

The data were analyzed in terms of hit RTs, miss rates, and false alarm counts. Data from one participant were not analyzed due to excessive false alarms (> 130), and data from one additional participant were not included in the hit RT analysis due to a 100% miss rate in one condition. For each dependent variable, we conducted a three-way repeated-measures ANOVA to study the effect of the relative-rate condition (more-color-target or more-orientation-target sessions), set size (4 or 12), and target type (color or orientation). Since we now have two session order variables (relative-rate session order and set-size session order), we did not include these variables in the analysis for simplicity. It was also less meaningful to include them at this point because each session order combination group had a relatively small sample size (~ 6). Similar to previous experiments, we conducted the corresponding Bayesian ANOVA to evaluate the possible null effects.

#### Hit RTs

The ANOVA and its Bayesian counterpart revealed a significant and decisive set-size effect [$F\left({1,23}\right)=41.29, p<.001, {\eta }_{p}^{2}=.64; {\text{BF}}_{{\rm inclusion}}>1000$ (slower for the larger set size)], a significant and decisive target-type effect [$F\left({1,23}\right)=36.13, p<.001, {\eta }_{p}^{2}=.61; {\text{BF}}_{{\rm inclusion}}>1000$ (slower for orientation targets)], and a significant and substantial interaction between set size and the target type [$F\left({1,23}\right)=12.08, p=.002, {\eta }_{p}^{2}=.34; {\text{BF}}_{{\rm inclusion}}=7.13$ (a larger set-size effect for orientation targets)]. These effects were trivial, which reflected an overall set-size effect. Since the orientation targets were visually less salient, they were associated with slower hit RTs and a less efficient search.

There was a significant but non-substantial interaction between relative-rate condition and target type [$F\left({1,23}\right)=5.52, p=.03, {\eta }_{p}^{2}=.19; {\text{BF}}_{{\rm inclusion}}=2.21$]. This result showed that color targets were detected more slowly when they were relatively rare [$t\left(23\right)=2.67, p=.01$], but this effect for orientation targets was less pronounced [$t\left(23\right)=0.63, p=.54$]. This pattern replicated that of Experiment 2.

**Fig. 6:**
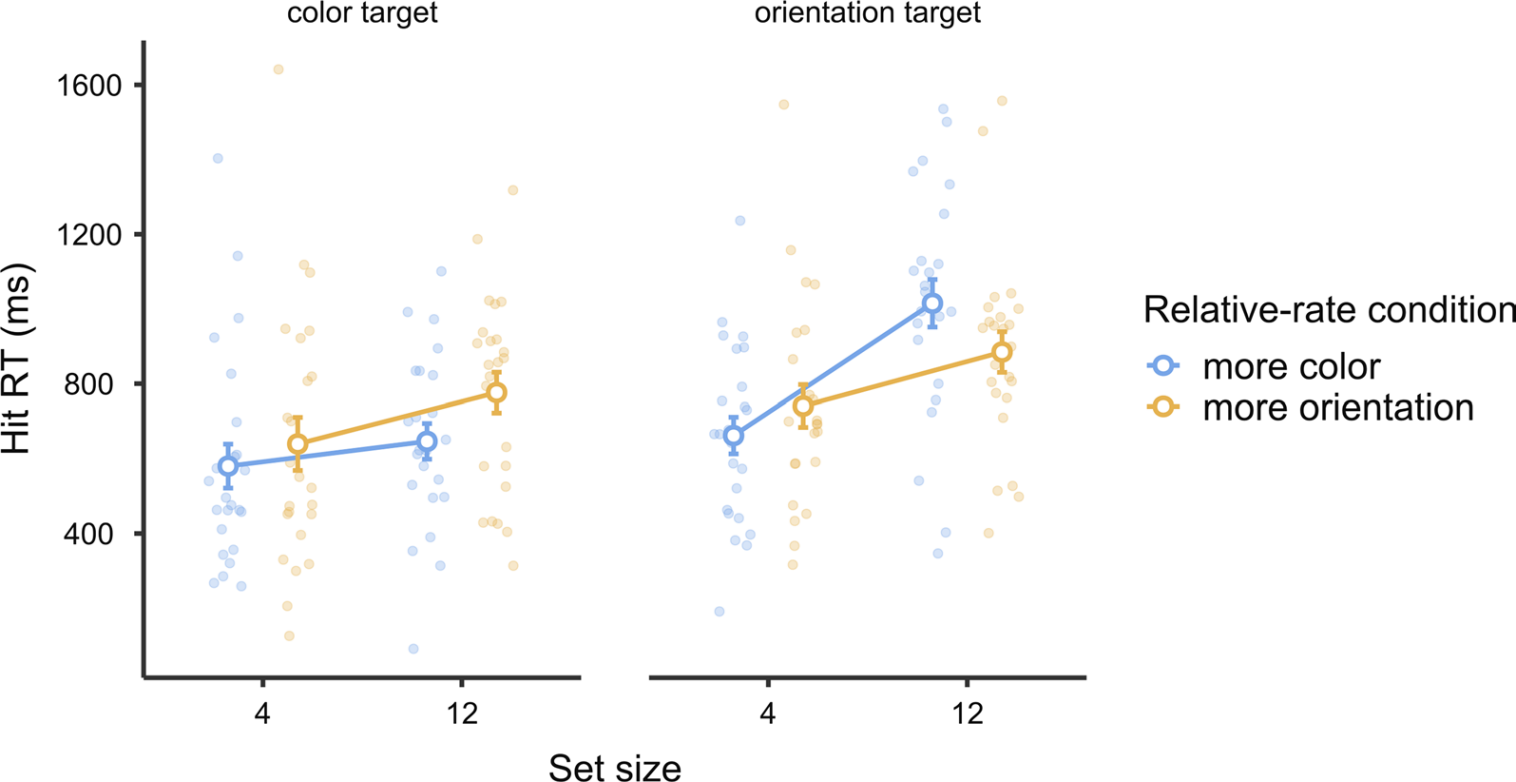
Experiment 3 results: hit RTs. *Note* In Experiment 3, a relative target-rate effect occurred in terms of set-size effects. The search tended to be more efficient (smaller set-size effect) when the current target type was more frequent. Error bars represent ± 1 standard error of the mean

In this experiment, we are most interested in the three-way interaction between the relative-rate condition, target type, and set size. This effect was statistically significant and substantial [$F\left({1,23}\right)=8.37, p=.008, {\eta }_{p}^{2}=.27; {\text{BF}}_{{\rm inclusion}}=7.54$]. This three-way interaction effect indicated that the search was more efficient when the relative-rate condition matched the target type (Fig. 6). In other words, attentional guidance was more effective when the current target type was relatively frequent. Breaking this effect down, concerning only orientation targets, the search was significantly and substantially more efficient in the more-orientation-target condition than in the more-color-target condition [$F\left({1,23}\right)=8.3, p=.008, {\eta }_{p}^{2}=.25; {\text{BF}}_{{\rm inclusion}}=7.07$]. Concerning only the color targets, the search was numerically more efficient in the more-color-target condition than in the more-orientation-target condition, but it did not reach statistical significance [$F\left({1,24}\right)=2.68, p=.12,{\eta }_{p}^{2}=.10; {\text{BF}}_{{\rm inclusion}}=0.50$]. There were no other significant effects in this ANOVA (all $p>.11$).

#### Miss Rate

**Fig. 7:**
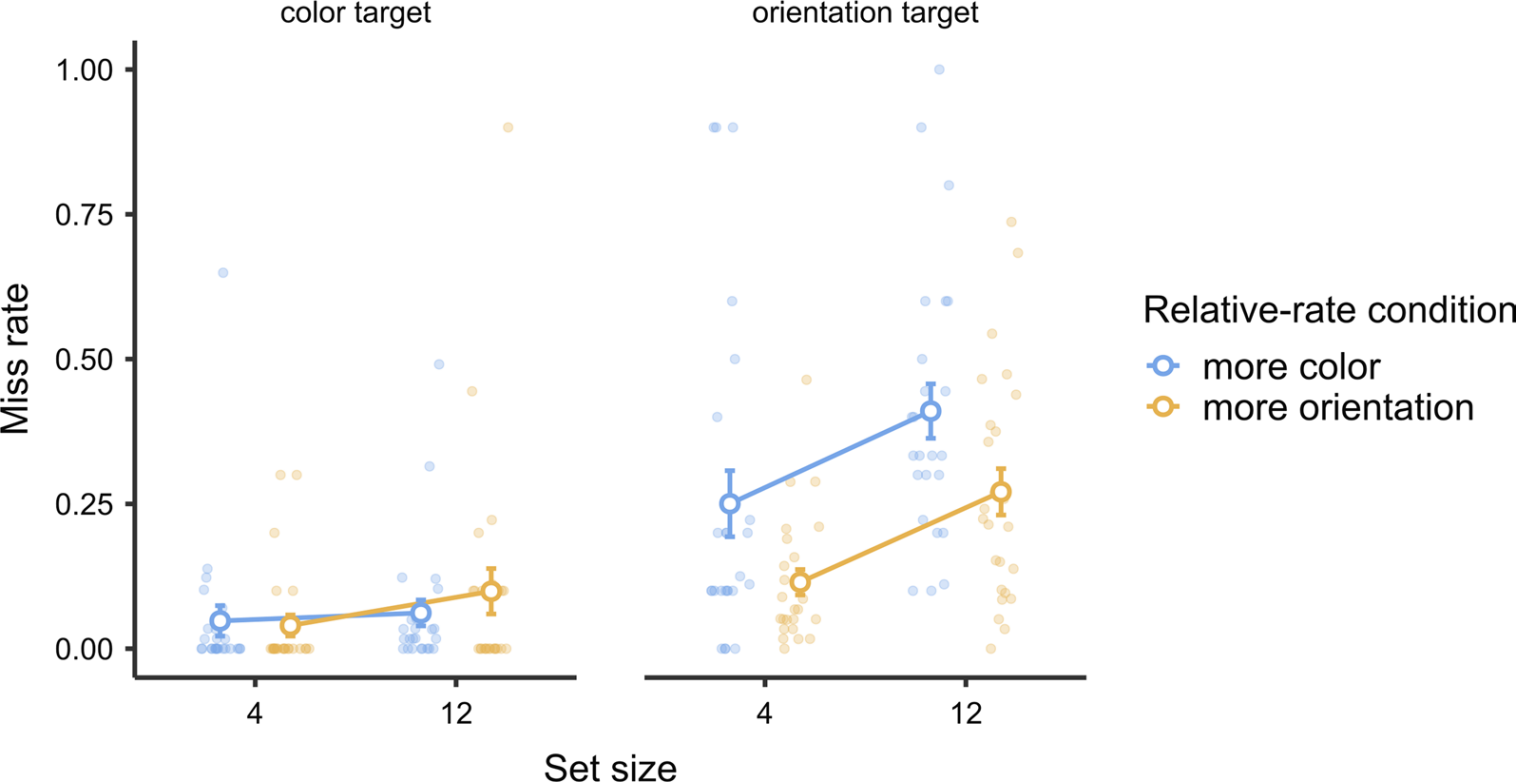
Experiment 3 results: miss rate. *Note* In Experiment 3, relative target-rate effects occurred in terms of miss rates for orientation targets only. For the color targets, there was an apparent floor effect. There were no relative target-rate effects on search efficiency. Error bars represent ± 1 standard error of the mean

The results of the ANOVA and its Bayesian counterpart showed a significant and strong relative-rate condition effect [$F\left({1,24}\right)=7.85, p=.01, {\eta }_{p}^{2}=.25; {\text{BF}}_{{\rm inclusion}}=16.28$ (more misses in more-color-target sessions)], a significant and decisive set-size effect [$F\left({1,24}\right)=37.28, p<.001, {\eta }_{p}^{2}=.61; {\text{BF}}_{{\rm inclusion}}>1000$ (more misses for a larger set size)], and a significant and decisive target-type effect [$F\left({1,24}\right)=90.49, p<.001, {\eta }_{p}^{2}=.79; {\text{BF}}_{{\rm inclusion}}>1000$ (more misses for orientation targets)]. As shown in Fig. 7, these effects were mainly driven by the orientation targets. The two-way interaction between target type and set size was significant and strong [$F\left({1,24}\right)=15.71, p<.001, {\eta }_{p}^{2}=.40;{\text{BF}}_{{\rm inclusion}}=25.68$], showing that the search was less efficient for the orientation targets.

The two-way interaction between relative-rate condition and target type was significant and decisive [$F\left({1,24}\right)=12.63, p=.002, {\eta }_{p}^{2}=.35; {\text{BF}}_{{\rm inclusion}}=360.15$], reflecting that more misses resulted when the current target type was relatively rare, consistent with our expectation. This effect was mainly driven by the orientation targets. For the orientation targets, there were significantly more misses in the more-color-target condition [$t\left(24\right)=4.36, p<.001$]. For the color targets, there was little difference between the two relative-rate conditions [$t\left(24\right)=0.49, p=.63$]. Since the miss rate of the color targets was quite low (6.4% overall), the lack of an effect with color targets probably reflected a floor effect (Fig. 7).

Regarding the main aim of this experiment, the three-way interaction between relative-rate condition, target type, and set size was not significant [$F\left({1,24}\right)=0.79, p=.38, {\eta }_{p}^{2}=.03;{\text{BF}}_{{\rm inclusion}}=0.32$]. This was not surprising because miss rate was not a sensitive measurement of attentional guidance.

There was no other significant effect in this ANOVA, and $p=.56$.

#### False alarms

**Fig. 8:**
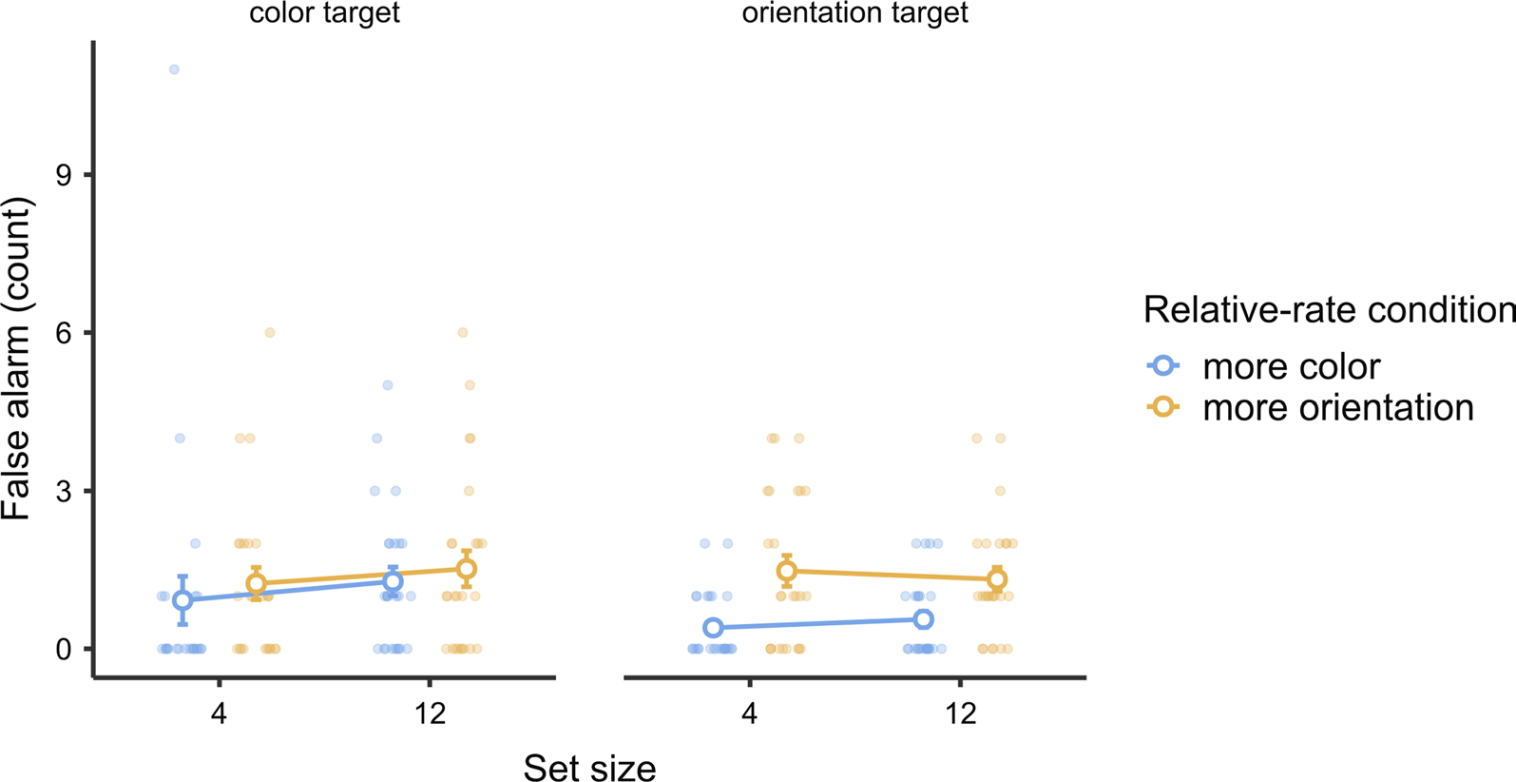
Experiment 3 results: false alarms. *Note* In Experiment 3, there were no relative target-rate effects in terms of false alarms. Error bars represent ± 1 standard error of the mean

An ANOVA and a Bayesian ANOVA on false alarms revealed a significant and strong relative-rate effect [$F\left({1,24}\right)=6.13, p=.02, {\eta }_{p}^{2}=.20; {\text{BF}}_{{\rm inclusion}}=14.04$], showing that there were more false alarms for the more-orientation-target conditions in general. We found no relative target-rate effect on false alarms, that is, there was no interaction between relative-rate condition and target types [$F\left({1,2}4\right)=1.67, p=.21, {\eta }_{p}^{2}=.07; {\text{BF}}_{{\rm inclusion}}=0.72$]. There were no other significant effects (all $p>.1$). The false alarm data are plotted in Fig. 8.

### Discussion

**Fig. 9:**
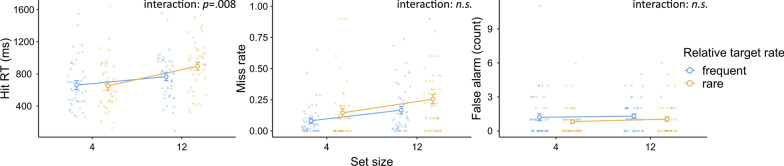
Experiment 3 results: data by relative frequency of the current target type. *Note* We rearranged the results by relative target frequency regardless of the current target type. As the graphs show, the search was significantly faster and more efficient in terms of hit RTs when the current target was frequent. There were significantly more misses for the rare target types, but they did not interact with set size. There was no effect on false alarms

We plotted the relative target-rate effects on hit RTs, miss rates, and false alarm counts by whether the target type was relatively frequent, collapsing across target types (Fig. 9). Generally, the results confirmed that there was a relative target-rate effect on hit RTs and set-size effects (on hit RTs) in continuous visual search. In other words, the search was faster and more efficient when the current target type was more frequent. As such, our finding was consistent with previous results in regular visual search (Godwin et al., 2015; Hout et al., 2015), where relative target prevalence affected the search via attentional guidance, at least for dissimilar targets. These results suggest that when a target type is relatively frequent, the searcher would prioritize the target features to make the search efficient, leading to a smaller set-size effect.

In addition to set-size effects, the current experiment also replicated a relative target-rate effect on miss rates. This confirmed the result of the last experiment, with a higher miss rate for the relatively rare target type. However, the relative target-rate effect on miss rates did not evolve to a set-size effect. This was reasonable because the miss rate was probably not a sensitive measure for set-size effects. In visual search literature, the set-size effects on RTs are coupled with misses quite usually, but we may not always expect them to reach statistical significance. Finally, similar to previous experiments, there were no relative target-rate effects on false alarms.

## Experiment 4

In the last experiment, although we demonstrated a relative target-rate effect on search efficiency in continuous visual search, it was unclear whether the target-rate effect on search efficiency generalizes from a relative manipulation to an absolute one. It is possible that the relative and absolute target rate (as well as target prevalence) effects were due to the different underlying processes. For example, whereas relative target-rate (and prevalence) effect on visual search may be more influenced by the relative distribution of attentional resources to finding each target, competition for attentional resources may not occur for absolute target rate (and prevalence) designs because only one target type was involved. From this perspective, one may not expect absolute target-rate manipulation to influence search efficiency. Any target-rate (or prevalence) effect may manifest mainly on the overall search time at a post-search stage. However, there are other possibilities. For example, it could be that seeing a target more frequently can help refine the target template representation, which facilitates attentional guidance and improves the search efficiency. Therefore, it is theoretically meaningful to test whether an absolute target-rate manipulation would influence search efficiency.

Another purpose of Experiment 4 was to directly test a vigilance account for the target-rate effect. Although we found relative target-rate effects in the last two experiments, where vigilance levels were controlled across target-rate manipulations, this only indicates that target-rate effects could occur without a vigilance difference. However, it does not preclude that an absolute target-rate manipulation may lead to a difference in vigilance level, contributing to the target-rate effect. Therefore, a direct test for how the target rate may influence vigilance is needed.

In Experiment 4, we manipulated the set size in an absolute design to ascertain whether there was any absolute target-rate effect on search efficiency. The search display was the same as that in Experiment 3 (Fig. 5), but the participants only had to detect the orientation targets. Color variations were irrelevant to the task. To evaluate the level of vigilance at each target rate, we also asked the participants to detect an occasional flash on the computer screen. The response time and accuracy of this flash detection task were interpreted to reflect the participants’ vigilance level.

### Method

#### Participants

A total of 28 undergraduate students from Hong Kong Baptist University and other local universities took part in the experiment with monetary compensation.

#### Apparatus and stimuli

The apparatus and stimulus were based on Experiment 3. However, only orientation targets were used. The item colors were changing as in previous experiments, although they were irrelevant to the task. The set sizes were 4 and 12, and their spatial arrangements were the same as those in Experiment 3. Similar to Experiments 2 and 3, the search items were presented on a white background. However, during a screen flash, an event that the participants had to detect in this experiment, the background was briefly dimmed to lch(85,0,0) for 50 ms.

#### Procedure

The procedure was based on previous experiments. The participants first received demo sessions that introduced the task and enabled them to practice the orientation search task, as well as the orientation search and flash detection dual task. The experiment would only proceed if the participant had performed satisfactorily in each demo session. The experiment proper contained four sessions, with two consecutive sessions for each target rate (frequent or rare), one for each set size (4 or 12). In the frequent target sessions, there were 42 targets and seven flashes. In the rare target sessions, there were seven targets and seven flashes. Each session lasted for seven minutes. Target-rate session order and set-size session order were counterbalanced across participants, similar to previous experiments.

The duration of each session was divided into target and flash intervals. These two interval types were randomly ordered with the constraint that the first interval must be a target interval. Each flash interval was 2 s long; as there were seven flashes in each session, the flash intervals accounted for 14 s of each session. The remaining time of each session was then equally divided among the target intervals. Within each target interval, at a random time point, an item would start changing into a target, similar to previous experiments. Each flash interval started with the search background turning 85% gray for 50 ms and then reverting to normal (white). In this experiment, the participants had to detect a vertical bar, ignore the colors, and press the “/” key as quickly and as accurately as possible in response. When a screen flash occurs, they must press the space bar in response as quickly and accurately as possible.

### Results: visual search

**Fig. 10:**
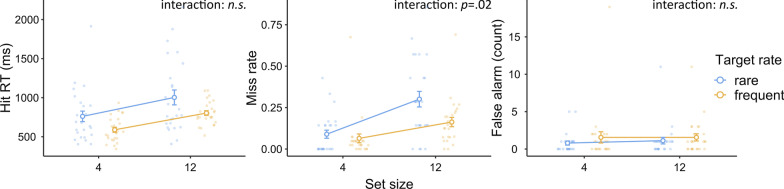
Experiment 4 results: visual search. *Note* In Experiment 4, target-rate effects occurred in terms of hit RT and miss rates but not on false alarms. Target rate did not affect search efficiency on hit RT, but there was an apparent set-size effect on miss rate. We believe the latter reflected a floor effect only. Error bars represent ± 1 standard error of the mean

The data were analyzed in terms of hit RTs, miss rates, and false alarm counts. Data from three participants were not analyzed due to excessive false alarms (> 80). For each dependent variable, we conducted a two-way repeated-measures ANOVA to study the effect of target rate (frequent or rare) and set size (4 or 12). Since we have two session order variables (target-rate session order and set-size session order), we did not include them in the analysis for simplicity. It was also less meaningful to include them as the sample size of each session order combination group was relatively small (~ 6). Similar to previous experiments, the corresponding Bayesian ANOVA was also conducted. The descriptive results are shown in Fig. 10.

#### Hit RTs

The ANOVA and its Bayesian counterpart revealed a significant and strong target-rate effect [$F\left({1,24}\right)=14.25, p<.001, {\eta }_{p}^{2}=.37; {\text{BF}}_{{\rm inclusion}}=74.99$ (slower for rare targets)] and a significant and decisive set-size effect [$F\left({1,24}\right)=20.43, p<.001, {\eta }_{p}^{2}=.46;{\text{BF}}_{{\rm inclusion}}=1111.06$ (slower for a larger set size)], which were well expected. However, there was a substantial inclination toward the lack of an interaction effect between target rate and set size [$F\left({1,24}\right)=0.08, p=.78, {\eta }_{p}^{2}=.00; {\text{BF}}_{{\rm inclusion}}=0.28$], indicating that although the participants searched slowly when the targets were rare, the corresponding set-size effect was not larger. Instead, the set-size effect was substantially the same across target-rate conditions.

#### Miss rate

The ANOVA and its Bayesian counterpart revealed a significant and strong target-rate effect [$F\left({1,24}\right)=13.11, p=.001, {\eta }_{p}^{2}=.35; {\text{BF}}_{{\rm inclusion}}=74.25$], indicating more misses when the target was rare. The set-size effect was also significant and decisive [$F\left({1,24}\right)=29.12, p<.001, {\eta }_{p}^{2}=.55; {\text{BF}}_{{\rm inclusion}}>1000$], indicating more misses with a large set size. Both effects were expected. The interaction effect between target rate and set size was significant but non-substantial [$F\left({1,24}\right)=6.68, p=.02, {\eta }_{p}^{2}=.22; {\text{BF}}_{{\rm inclusion}}=2.34$]. This effect showed that the target-rate effect on miss rate was more visible at a higher set size. At a small set size, the miss rate was quite low at both target rates (rare: 9%, frequent: 6%). Thus, the lack of a target-rate effect at a small set size could reflect a floor effect.

#### False alarms

The ANOVA on false alarm counts revealed no statistically significant effects (all $p>.2$). The Bayesian ANOVA substantially favored not including a set-size effect (${\text{BF}}_{{\rm inclusion}}=0.22$) or a target-rate × set-size interaction effect (${\text{BF}}_{{\rm inclusion}}=0.30$). Its inclination toward a null target-rate effect was non-substantial (${\text{BF}}_{{\rm inclusion}}=0.49$).

### Results: detection of screen flashes

**Fig. 11:**
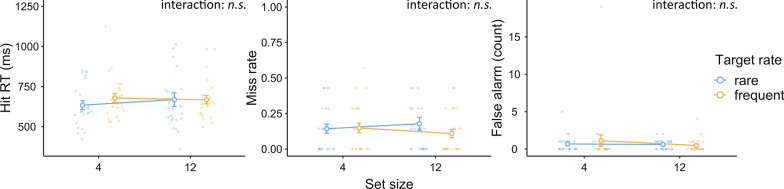
Experiment 4 results: flash detection. *Note* In Experiment 4, we monitored the participants’ vigilance levels by asking them to detect occasional screen flashes. There were no significant target-rate effects on flash detection in terms of hit RT, miss rates, or false alarms. Error bars represent ± 1 standard error of the mean

To understand the level of general vigilance at each target rate, we measured the participants’ RTs to screen flashes. Data were analyzed with a two-way repeated-measures ANOVA and Bayesian ANOVA. The descriptive results are shown in Fig. 11.

#### Hit RTs

Data from one more participant were not included due to a 100% miss rate in some conditions. The ANOVA revealed no effects of target rate, set size, or their interaction (all $p>.3$). The Bayesian analysis favored the lack of these effects non-substantially, substantially, and non-substantially at ${\text{BF}}_{{\rm inclusion}}=0.32$, ${\text{BF}}_{{\rm inclusion}}=0.24$, and ${\text{BF}}_{{\rm inclusion}}=0.43$, respectively.

#### Miss rate

There were no statistically significant effects in the ANOVA on-screen flash detection miss rates (all $p>.2$). The Bayesian analysis favored the lack of a target-rate, set-size, and target-rate × set-size interaction effect non-substantially, substantially, and non-substantially at ${\text{BF}}_{{\rm inclusion}}=0.34$, ${\text{BF}}_{{\rm inclusion}}=0.21$, and ${\text{BF}}_{{\rm inclusion}}=0.52$ , respectively.

#### False alarms

There were no statistically significant effects in the ANOVA on false alarm counts (all $p>.3$). The Bayesian analysis favored the lack of a target-rate, set-size, and target-rate × set-size interaction effect non-substantially, substantially, and substantially at ${\text{BF}}_{{\rm inclusion}}=0.49$, ${\text{BF}}_{{\rm inclusion}}=0.22$, and ${\text{BF}}_{{\rm inclusion}}=0.30$, respectively.

### Discussion

There were two main findings in Experiment 4. First, both target rate and set size affected the hit RTs and miss rates in the visual search task. Rare targets and a larger set size led to slower detection and more misses. This is consistent with previous experiments and previous regular visual search results. However, contrary to Experiment 3, we found no target-rate effect on search efficiency (on hit RTs), and the Bayesian analysis confirmed the lack of this effect. This result indicates that relative and absolute target rates might influence the search via different mechanisms. In relative target-rate experiments, the participants had to search for two target features at the same time. Therefore, how they allocate their attentional resources to each target might be critical to the search performance. For instance, when setting up top-down guidance for two search targets, the relative target rate could influence how much the current attentional set was biased toward each target. The effectiveness of attentional guidance to each target would then be reflected in terms of set-size effects. However, in absolute target-rate experiments, there was no such resource competition between two targets. Whether a target was presented frequently or rarely, the current attentional set should simply be optimized toward the only target type. Therefore, the set-size effects remained unaltered.

Another main finding of this experiment was a lack of target-rate effect on-screen flash detection, whether in terms of hit RTs, miss rates, or false alarms. This finding goes against the idea that a higher target rate makes participants to be more vigilant or alert in general and leads to better search performance.

Taken together, our findings suggest that the absolute target rate did not influence search performance via attentional guidance or vigilance level. Therefore, it seems that the absolute target-rate effect in continuous visual search mainly arises from a post-search stage.

## General discussion

The purpose of this study was to broaden the ongoing investigation on visual search from a regular trial-by-trial variant to a continuous one; both search types are common in real-life scenarios. We began this investigation by designing a continuous visual search task that is easy to manipulate and provides a good abstraction of its real-life counterparts. In our continuous search task, people looked for one or two target features (color, orientation, or both) among items with varying colors and/or orientations over sessions of several minutes. There were no separate trials within each session. We manipulated the target rates (i.e., the occurrence frequency of the target features) and studied whether they affected continuous visual search in similar ways to the LPE in regular, discrete visual search.

In the four experiments we conducted, we found evidence for a target-rate effect in continuous visual search. In Experiment 1, slower hit RTs and higher miss rates were observed when a target was rare. In Experiment 2, when searching for two target features, slower hit RTs and higher miss rates were associated with a relatively rare target feature. In Experiment 3, a larger set-size effect was found for a relatively rare target feature, showing that relative target rate influenced search performance via the effectiveness of attentional guidance. In Experiment 4, it was found that set-size effects were not affected by absolute target-rate manipulations, indicating that only relative target-rate manipulations would influence search efficiency. Furthermore, using a flash detection paradigm, we showed that the absolute target rates did not influence vigilance in continuous visual search.

Our results provided an initial evaluation of the possible explanations for the target-rate effect in continuous visual search. First, the presence of target-rate effects in relative prevalence designs and their absence in flash detection performance rejected a general vigilance explanation. Second, search efficiencies in terms of set-size effects were specifically influenced by relative target-rate manipulations. This indicated that the relative target-rate effect was associated with better attentional guidance to the relatively frequent targets. Third, our data cannot be explained by target exposure history, habitual response, or premature search termination. Taken together, in single-target scenarios, the target-rate effect most likely occurred at a post-search target identification stage. In multiple target scenarios, the target-rate effect was also due to the strategic allocation of attentional resources to each target feature.

### Generalization of the LPE to continuous visual search

The main finding of the current study is a target-rate effect in continuous visual search, which is similar to the target-prevalence effect known previously for regular visual search. This finding supports the generalization of knowledge and the associated precautions from regular visual search to continuous visual search. Targets being rare has been raised as a real-life challenge in continuous visual searches like lifeguarding (Lanagan-Leitzel et al., 2015) or face searches in CCTV surveillance (Mileva & Burton, 2019). However, no previous studies have experimentally tested how previous knowledge on regular visual search is generalized to continuous visual search. The current study provides a piece of direct evidence for such a generalization.

The current results also suggest that the explanations for the target-rate effect in continuous visual search are similar to the LPE in regular search. Consistent with previous suggestions that regular search LPE mainly comes from a post-search target identification stage (Godwin et al., 2015; Hout et al., 2015), we reached the same conclusion in this study. Previous visual search and vigilance studies generally suggest that target prevalence influences target identification via a decision bias. In visual search, it is believed that a low target prevalence biases the participant’s response criterion (c) toward target-absent responses without significantly reducing the perceptual sensitivity (d’) to the target (Wolfe & Van Wert, 2010). In vigilance tasks, the rate of a target event (number of targets per unit time) can be decomposed into the event rate (number of events per unit time) and signal probability (number of targets per a fixed number of events). A high event rate is known to cause vigilance decrement (increasing misses over time) by biasing the participant’s criterion toward an absent decision (Mackworth, 1965), unless when the event rate is very high, it also leads to a perceptual challenge (Parasuraman, 1979). A low signal probability is known to increase the miss rate, similar to the LPE in visual search. Colquhoun (1961) found that this effect is due to a decision bias. Therefore, we hypothesize that continuous visual search shares a similar locus to prevalence effects on regular visual search and vigilance tasks, that is, target frequencies seem to cause a decision bias in all cases.

### Absolute versus relative target-rate effects

In addition to the basic target-rate effect, another major result of this study is a relative target-rate effect in continuous visual search, which conceptually replicated the relative LPE in regular search (Godwin et al., 2015; Hout et al., 2015; Wolfe et al., 2007). Such a finding was important in demonstrating that the LPE cannot be “cured” by increasing the overall target prevalence with non-critical targets. Instead, the relatively rare targets were still missed more often than the relatively frequent targets (Wolfe et al., 2005, 2007). The current study demonstrated the same phenomenon in continuous search. For instance, although it is not possible to directly compare absolute and relative target-rate conditions among our different experiments due to unmatched stimulus parameters, a rough comparison of the average figures may still be informative. In Experiments 1 and 4, the absolute target-rate produced effects of 7.2 and 8.2 percentage points on miss rates, whereas in Experiments 2 and 3, the relative target-rate effects were 12.1 and 7.6% points on miss rates. In general, the relative target-rate effects were no weaker than the absolute target-rate effects. Therefore, including non-rare targets in continuous visual search does not “cure” and may even worsen the rare target effect.

Another downside of “curing” rare target effects by including non-rare targets is a potential dual-target cost (Menneer et al., 2007, 2010). For instance, top-down guidance must be less optimal when configured for two target representations than one, leading to poorer search performance. We observed such costs in our experiments. Comparing Experiment 1 (single-target) with Experiment 2 (dual-target), the miss rates for the color targets were higher in the latter (35.5%) than the former (12.5%), with $t\left(67\right)=5.26$ and $p<.001$, even though Experiment 2 used a smaller set size and a higher contrast from the background, which should theoretically lead to better performance. Comparing Experiment 3 (dual-target) with Experiment 4 (single-target), the average miss rates for the orientation targets were higher in the former (26.2%) than the latter (15.4%), with $t\left(48\right)=2.46$ and $p=.02$. The set size and the stimulus parameters were the same across Experiments 3 and 4. Therefore, we observed a dual-target cost in our experiments, that is, dividing attention across two target features leads to poorer overall performance in continuous search.

### Attentional guidance and target-rate effects

A theoretically interesting question to ask about the target-rate effects in continuous visual search, as well as LPEs in regular search, is whether target rates influence search performance via the effectiveness of attentional guidance. If better attentional guidance was associated with more frequent targets, we should observe more efficient searches as a result. We found such evidence in Experiment 3 with a relative target-rate manipulation, but not in Experiment 4 with an absolute manipulation. We reasoned that it was because target rate only influences attentional guidance when two targets compete for attentional resources.

Our findings were consistent with previous findings in regular visual search. For example, with absolute designs, Wolfe et al.’s (2005) original study did not observe any LPE in terms of set-size effects in target-present trials. Wolfe et al. (2007) did not observe such effects either. Second, with relative designs, Hout et al.’s (2015) and Godwin et al.’s (2015) eye movement data showed shorter first-landing times for the more frequent targets, indicating better attentional guidance to them.

Theoretically, if attentional guidance is better with more frequent targets in relative prevalence designs, we should also expect a higher search efficiency. However, previous studies that manipulated set size in relative prevalence designs did not observe any LPE in terms of search efficiency in target-present trials (Menneer et al., 2010; Wolfe et al., 2007). One factor that may explain the lack of effects in these studies is the search stimulus. For instance, Menneer et al. (2010) used conjunctively defined colored shapes, while Wolfe et al. (2007) used X-ray-like baggage images; both studies used non-salient stimuli. Such stimulus choices might have rendered attentional guidance ineffective at all prevalence levels, undermining any LPE on search efficiency. However, in their studies, Hout et al. (2015) and Godwin et al. (2015) used simple objects or shapes that allowed the targets to be distinguished from the distractors in terms of basic color and shape. As a result, the participants can develop effective attentional guidance based on basic features, providing enough room for target prevalence to exert its influence.

### Two types of continuous search

An aim of the current study was to generalize previous research findings to broader search scenarios. We examined an instance of continuous search, in which observers looked for an occasionally occurring feature among other items with varying non-target features. This laboratory task abstracts the key features of real-life search tasks. Take lifeguarding as an example. Lifeguards have to look for specific movement patterns (drowning) among other less important movement patterns (swimming, playing, diving, etc.). In this type of continuous search, the target item possesses the target feature only temporarily. In our experiments, the color or orientation of a target item only stopped at the target state for 2 s and then reverted to the normal state afterward to become a non-target again. In lifeguarding, the drowning movement may only last for a few minutes.

We believe there is another type of continuous search that should also be examined experimentally. In this type of continuous search, the target item permanently possesses the target feature, but the search items are themselves temporary because they move in and out of the monitoring area. This type of search covers practical scenarios, such as infrared body temperature surveillance, in which the security officer has to detect persons with elevated body temperature (as coded in color) before they leave the screen (the monitoring region). Another example is a police officer who might need to search for a specific person by monitoring a CCTV display, while people enter and leave the monitored area. The police officer has to identify the target person before that person leaves the screen.

In general, we hypothesize that these two types of continuous search are similar in most regards, as they share key common features, such as dynamic displays, multiple items, and temporary targets. In fact, the preliminary data in our laboratory demonstrated an expected target-rate effect on hit RTs and a miss rate in searches of the second kind. Nevertheless, the two types of searches are not without differences. For example, in the second kind of continuous search, the search items typically enter the display from an edge of the monitoring area rather than appearing from nowhere. Therefore, if there is a general item flow direction (such as pedestrians entering the screen mostly from one side), it is intriguing to see whether more attention may be allocated to the entering edge of the screen or not.

### Previous studies on visual search with dynamic properties

While most previous studies used static stimuli in visual search, there were attempts to study visual search with dynamic properties. For example, Laxton and Crundall (2018) compared lifeguard and non-lifeguard visual search performances using video clips of confederate swimmers. Meanwhile, Mileva and Burton (2019) studied visual search for faces obtained from photo ID or social media in CCTV surveillance video clips. However, neither study manipulated target prevalence. Furthermore, these studies differed from the current research as they divided the search into 30-s trials, and each trial could only contain zero or one target. Therefore, the decision process involved in such experimental design may be more akin to a regular visual search. For example, by knowing that there would be at most one target in any given trial, you may stop searching upon the detection of a target, you may give up searching when you feel that a target is not going to appear after inspecting most of the video, or when you are unsure about whether a target exists (e.g., you missed part of a trial due to an attention lapse), you may still make an educated guess based on your knowledge of target prevalence (Schwark et al., 2013). Therefore, there is generally more room for an observer to determine their response strategically in a trial-by-trial dynamic search with zero or one target than in a continuous visual search. By contrast, in a continuous search, the number of targets is generally unknown. In such a case, less information is available to support a strategic response. Therefore, the decision process between the two types of dynamic search could be very different.

A more relevant recent study that used dynamic stimuli was conducted by Muhl-Richardson et al. (2018). Not only did they manipulate target prevalence in their study, but they also used a more variable number of targets in their dynamic search trials. They asked the participants to search for a cued target color among an array of 108 color-changing squares. Each trial lasted for 40 s and may contain 0, 1, or 2 targets. The purpose of their study was to investigate whether people detected the target color in a predictive fashion. To achieve this purpose, while most distractors would not get close to the target color, they let some distractors get close to (but not reach) the target color. By recording eye movements, they found that the participants fixated on these target-similar distractors, which is indicative of predictive search behavior.

The third experiment in Muhl-Richardson et al.’s (2018) study was most relevant to our purpose. They manipulated target prevalence and unexpectedly found no target prevalence effect in terms of miss rate and RTs. There were more false alarms in their low prevalence condition, which was quite uncommon in previous studies. Intuitively, a low target prevalence should be associated with fewer false alarms. They commented that perhaps these discrepant findings were due to differences between static and dynamic searches. However, as opposed to this speculation, our current findings clearly suggest that a target-rate effect can occur in a continuous and dynamic visual search. Thus, an alternative explanation of their results is warranted. To explain their findings, we believe the critical uniqueness of their task was their use of target-similar distractors. For instance, the relatively high false alarm rate in their experiments (~ 30%) was likely caused by these target-similar distractors. In their high-prevalence condition, the false alarm rate reduced to ~ 20%, while in their low prevalence condition, it increased drastically to ~ 60%. We believe this may reflect that when there were more targets in the experiment, there might be a higher chance for the participants to learn to discern them from target-similar distractors. Therefore, while a high false alarm rate in most other studies may reflect a liberal decision criterion, a high false alarm rate in Muhl-Richardson et al.’s study may mainly reflect the perceptual confusion between the target and the target-similar distractors. In our experiment, however, we have a relatively wide feature zone reserved for the target. Thus, distractors and targets are unlikely to get confused. As such, we observed similar target-rate effects to regular visual search.

### Continuous visual search and event rates

The most important finding in vigilance literature was that vigilance performance drops over time, and a high event rate would increase such decrement (e.g., Mackworth, 1965). In vigilance tasks, the target rate and event rate are related but have distinct manipulations (Colquhoun, 1961; Mackworth, 1965). Previous studies have generally shown that event-rate and target-rate effects in vigilance tasks are mostly results of decision bias, except for very high event rates in which perceptual limitations may come into play (Parasuraman, 1979). In visual search literature, event rates were rarely systematically manipulated; in our experiments, they were not manipulated either. However, event rates could influence visual search in a similar manner, which warrants further research. For example, we may want to avoid a too-high event rate in visual search if it causes perceptual constraints. This way, the issue of event rate would arguably be more critical for continuous search compared with regular, discrete visual search. In real-life applications of discrete search, the event rate can often be controlled by the searcher (e.g., a security officer can stop at a certain piece of baggage for more careful inspection, or a radiologist can inspect a medical image one after another on his/her own pace). However, in real-life continuous search, the event rates may not be controlled by the searcher. For example, the security officer cannot control how fast people move in and out of the surveillance region, and the lifeguard cannot control the speed with which patrons swim at the beach. Therefore, the only way to avoid a high event rate from going too high is to plan ahead on the resource management of the business. For example, one may reduce the event rate by recruiting more searchers and dividing the monitoring regions. Future research on the nature and behavior of event-rate effects is important, especially for continuous visual search.

### Target-rate effects on false alarms

In general, the current results indicated a target-rate effect on continuous visual search. Rare targets led to slower hit RTs and more misses. According to previous research on vigilance and visual search, decision bias may be a major cause of target-rate effects. However, according to this explanation, not only would a low target rate lead to a higher miss rate, but a high target rate should also lead to a more liberal criterion in accepting targets, with more false alarms ensuing. However, there were no target-rate effects on false alarms in our data. Instead, in all our experiments, the Bayesian analysis favored a lack of target-rate effect on false alarms, especially in Experiments 1 and 2.

We postulate three possible reasons for the lack of a target-rate effect on false alarms. The first possible reason is the floor effect. In our experiments, the number of false alarms per session was not high (3.8, 6.4, 2.2, and 1.3, respectively, for each experiment; the session durations were 15, 15, 10, and 7 min, respectively). However, they were not too close to zero either. Therefore, we are unsure how much our false alarms reflected unsystematic motor errors and how much they reflected genuine decision errors. We suppose there may be a mild floor effect, but it should not obscure the target-rate effect on false alarms altogether.

The second possibility is related to the way we analyzed the false alarm results. For instance, since continuous search is not separated by trials, we reported false alarm *counts* in our experiments because we do not have well-defined background events for calculating a false alarm *rate* like in regular discrete search. In discrete search studies, false alarm rates were calculated relative to the number of target-absent trials. As such, with the same false alarm count, the false alarm rate would be higher in a high-prevalence condition, where there were fewer target-absent trials. In continuous visual search, although the background events for false alarms to occur were ill-defined, they nevertheless occurred (i.e., the changing of non-target colors and orientations). Obviously, this should be taken into account in the analysis of false alarms, and it may not be correct to directly compare false alarm counts across target-rate conditions. It is possible that our lack of effect on false alarms is an illusion due to this incorrect comparison. Imagine dividing a search session into many imaginary time slices, and consider each time slice to be analogous to a search trial in a regular search task. If there was a target in a time slice, it was a “target-present slice”; otherwise, it was a “target-absent slice.” In this case, there would be more target-absent slices in a low-prevalence session. As such, if we calculate the false alarm “rate” relative to the number of “target-absent slices,” theoretically, the same false alarm count should result in a lower false alarm rate in a low-prevalence session and vice versa, consistent with the usual expectation.

The third possibility is related to a possible difference in the decision process between regular and continuous visual search. In regular visual search, Schwark et al. (2013) demonstrated that the search was driven by at least two types of decisions: search-based and prevalence-based decisions. A search-based decision means the observer finds and sees the target and then makes a target-present response. This decision is based on perception. Prevalence-based decision, on the other hand, is a cognitive one. For example, when the target prevalence is very high, the observer may think that it is a good bet to make a target-present response even if the target is not clearly seen. To distinguish these two kinds of decisions, Schwark et al. asked the participants to click on the target if they detected a target, but they also allowed the participants to press a target-present button (TP button) if they want to make a target-present response without locating the target. The TP button here represents the case wherein a response was made strategically, but not perceptually. It was found that the TP button was used more in higher prevalence and more difficult searches, providing evidence for prevalence-based decisions. Therefore, target prevalence may influence search performance in two ways. First, it may influence the search process. Wolfe and Van Vert (2010) suggested that target prevalence may alter our decision criterion when we inspect each search item during a search, leading to false detection or overlooking of targets. Second, it may influence strategic decisions. In prevalence-based decisions, even without perceiving any item as a target (whether it is, in fact, a target or not), one may still make a target-present response as a bet.

This distinction may be related to our lack of a false alarm effect. In our continuous visual search experiments, the search process is generally similar to regular searches. The observer inspects different search items throughout a search session, and, occasionally, the observer may accept some search items as targets, leading to detection responses. Target-rate manipulations may influence search-based decisions by changing the decision criterion used in each item inspection. However, continuous visual search is different from regular visual search because it is not separated by trials. It is reasonable to assume that prevalence-based decisions take individual trials as decision units. When making a prevalence-based decision, the cognitive process in an observer may be as follows: “I am not sure if there is a target *in this trial*, but since a target existed in *most of the trials*, let me make a target-present response *for this trial.*” However, this decision process may not be applied to a continuous visual search, where the observer sits and waits for a target without being forced to make a decision for each trial. For this reason, prevalence-based decisions may be much less common in continuous visual search. As such, in continuous visual search, we may expect that while the part of target-rate effects on false alarms attributed to search-based decisions may be preserved, the part due to prevalence-based decisions may diminish. Critically, based on the results of Experiment 1 in Schwark et al.’s study (2013, Fig. 5), whereas search-based detections (target click responses) were highly accurate, most false alarms actually came from prevalence-based detections (TP button responses). If it is generally true that most false alarms in visual search are due to prevalence-based decisions and that prevalence-based decisions are extremely reduced in continuous visual search, then it is reasonable to see a lack of target-rate effect on false alarms in continuous visual search.

### The decision process

As mentioned above, a critical difference between regular and continuous visual search lies in their response choices, and this difference requires us to reconsider the approach to model the associated decision processes. In regular trial-by-trial visual search, one chooses between three possible responses at any moment: target present, target absent, and continue searching. A popular approach to model this decision is to view it as a diffusion process of gathering and gauging statistical evidence for or against the presence of a target. Should it reach the response criterion of either decision, a corresponding response could be made; otherwise, the search continues. In diffusion models, statistical evidence is relative; technically, it represents the (log) odds of one response over the other. Thus, in the realm of discrete visual search, the presence of a perceptual signal is statistical evidence for a target-present trial, whereas the lack of a perceptual signal is statistical evidence for a target-absent trial. In other words, diffusion models can integrate the fact that you have waited long enough but did not succeed in spotting any targets as a good sign of a target-absent trial.

However, in continuous visual search, the task is not to gauge evidence between target-present and target-absent responses. The lack of a target signal here is an uninteresting baseline state, which does not constitute any responses (no matter how long you rested in it). This analysis is true, at least when the number of targets in a session is unknown. On the other hand, there is only a target-present response, and it should be triggered upon the detection of a signal. This situation is more akin to the case of the signal detection theory (SDT), in which the decision criterion is a level of perceptual signal strength rather than the odds between two possible situations. Thus, one discerns whether an input is more likely to be a signal or a noise by comparing it with critical signal strength.

Although it seems that the SDT is a more suitable approach than diffusion models in understanding the decision process involved in continuous search, it should be noted that the current data analysis practices for SDT cannot be directly applied to continuous search. In SDT, researchers typically analyze hit, miss, correct rejection, and false alarm data and then transform them into d’ (sensitivity) and c (response criterion) measures. In continuous search, we do not have well-defined numbers of correct rejections, and false alarms cannot be expressed as a ratio between the number of false alarms and negative trials. In our experiments, we focused our analysis on hit RT and miss rates. Further theoretical work would be necessary to determine the best way to measure the sensitivity and response criteria in the continuous domain.

## Conclusion

In our everyday lives, continuous visual search is no less common than discrete visual search. Many visual surveillance tasks take a form of continuous visual search that is not well modeled by the more simplified vigilance tasks that attracted research interest back in the 1950s. Therefore, the current study attempts to address this research gap by designing a simplified paradigm that captures the key characteristics of continuous visual search. We began our investigation by looking for a target-rate effect, which is roughly equivalent to the LPE in a regular visual search. In general, we found that more misses and slower hit RTs were associated with a lower target rate, similar to the LPE. We hypothesize that this effect originates from a more conservative decision criterion with rare targets. When asked to look for multiple targets, a higher miss rate and slower hit RTs were associated with the relatively rare target compared with the relatively frequent target. The relative target-rate effect was also observed in terms of search efficiency, which reflected a competition between the frequent and rare target features when spatial attention is guided to them. We conclude that previous findings in regular visual search generally apply to continuous visual search, and the main difference between regular and continuous visual search lies in their decision process. Future research should examine more variants of continuous visual search and develop data analysis methods to suit the distinctive needs of visual search of this kind.

## Data Availability

Data analysis of this study is available on the Open Science Framework at https://osf.io/g8kq4/

## References

[CR1] Anderson BA (2014). On the precision of goal-directed attentional selection. Journal of Experimental Psychology: Human Perception and Performance.

[CR2] Colquhoun WV (1961). The effect of ‘unwanted’ signals on performance in a vigilance task. Ergonomics.

[CR3] Drury, C. G., Green, B. D., & Lin, J. F. (2007). Fatigue in aviation inspection: Laboratory and validation studies. In *Contemporary ergonomics 2007* (pp. 41–46). Taylor & Francis.

[CR4] Drury C, Hoffman R, Hancock P, Scerbo M, Parasuraman R, Szalma J (2015). Sustained attention in operational settings. The Cambridge Handbook of applied perception research (Cambridge Handbooks in Psychology).

[CR5] Fleck MS, Mitroff SR (2007). Rare targets are rarely missed in correctable search. Psychological Science.

[CR6] Ghylin KM, Drury CG, Batta R, Lin L (2007). Temporal effects in a security inspection task: Breakdown of performance components. Proceedings of the Human Factors and Ergonomics Society Annual Meeting.

[CR7] Godwin HJ, Menneer T, Riggs CA, Cave KR, Donnelly N (2015). Perceptual failures in the selection and identification of low-prevalence targets in relative prevalence visual search. Attention, Perception and Psychophysics.

[CR8] Horowitz TS, Cade BE, Wolfe JM, Czeisler CA (2003). Searching night and day: A dissociation of effects of circadian phase and time awake on visual selective attention and vigilance. Psychological Science.

[CR9] Hout MC, Goldinger SD (2015). Target templates: The precision of mental representations affects attentional guidance and decision-making in visual search. Attention, Perception, & Psychophysics.

[CR10] Hout MC, Walenchok SC, Goldinger SD, Wolfe JM (2015). Failures of perception in the low-prevalence effect: Evidence from active and passive visual search. Journal of Experimental Psychology: Human Perception and Performance.

[CR11] Kunar MA, Watson DG (2011). Visual search in a multi-element asynchronous dynamic (MAD) world. Journal of Experimental Psychology: Human Perception and Performance.

[CR12] Kunar MA, Watson DG (2014). When are abrupt onsets found efficiently in complex visual search? Evidence from multielement asynchronous dynamic search. Journal of Experimental Psychology: Human Perception and Performance.

[CR13] Lanagan-Leitzel LK, Skow E, Moore CM (2015). Great expectations: Perceptual challenges of visual surveillance in lifeguarding. Applied Cognitive Psychology.

[CR14] Laxton V, Crundall D (2018). The effect of lifeguard experience upon the detection of drowning victims in a realistic dynamic visual search task. Applied Cognitive Psychology.

[CR15] Mackworth JF (1965). Deterioration of signal detectability during a vigilance task as a function of background event rate. Psychonomic Science.

[CR16] Mackworth NH (1948). The breakdown of vigilance during prolonged visual search. Quarterly Journal of Experimental Psychology.

[CR17] Mathôt, S. (2017). Bayes like a Baws: Interpreting Bayesian repeated measures in JASP. In *Cognitive Science and more*. Retrieved from: https://www.cogsci.nl/blog/interpreting-bayesian-repeated-measures-in-jasp.

[CR18] Menneer T, Barrett DJ, Phillips L, Donnelly N, Cave KR (2007). Costs in searching for two targets: Dividing search across target types could improve airport security screening. Applied Cognitive Psychology.

[CR19] Menneer T, Donnelly N, Godwin HJ, Cave KR (2010). High or low target prevalence increases the dual-target cost in visual search. Journal of Experimental Psychology: Applied.

[CR20] Mileva M, Burton AM (2019). Face search in CCTV surveillance. Cognitive Research: Principles and Implications.

[CR21] Molloy R, Parasuraman R (1996). Monitoring an automated system for a single failure: Vigilance and task complexity effects. Human Factors.

[CR22] Muhl-Richardson A, Godwin HJ, Garner M, Hadwin JA, Liversedge SP, Donnelly N (2018). Individual differences in search and monitoring for color targets in dynamic visual displays. Journal of Experimental Psychology: Applied.

[CR23] Nagy AL, Sanchez RR (1990). Critical color differences determined with a visual search task. Journal of the Optical Society of America A.

[CR24] Parasuraman R (1979). Memory load and event rate control sensitivity decrements in sustained attention. Science.

[CR25] Rich AN, Kunar MA, Van Wert MJ, Hidalgo-Sotelo B, Horowitz TS, Wolfe JM (2008). Why do we miss rare targets? Exploring the boundaries of the low prevalence effect. Journal of Vision.

[CR26] Schwark JD, MacDonald J, Sandry J, Dolgov I (2013). Prevalence-based decisions undermine visual search. Visual Cognition.

[CR27] The jamovi project (2022). *jamovi* (Version 2.2) [Computer Software]. Retrieved from https://www.jamovi.org

[CR28] Thomson DR, Smilek D, Besner D (2015). Reducing the vigilance decrement: The effects of perceptual variability. Consciousness and Cognition.

[CR29] Treisman AM, Gelade G (1980). A feature-integration theory of attention. Cognitive Psychology.

[CR30] Wolfe JM (1994). Guided search 2.0 a revised model of visual search. Psychonomic Bulletin and Review.

[CR31] Wolfe JM, Pashler HE (1998). Visual search. Attention.

[CR32] Wolfe JM (2016). Use-inspired basic research in medical image perception. Cognitive Research: Principles and Implications.

[CR33] Wolfe JM (2020). Visual search: How do we find what we are looking for?. Annual Review of Vision Science.

[CR34] Wolfe JM, Cave KR, Franzel SL (1989). Guided search: An alternative to the feature integration model for visual search. Journal of Experimental Psychology: Human Perception and Performance.

[CR35] Wolfe JM, Horowitz TS, Kenner NM (2005). Rare items often missed in visual searches. Nature.

[CR36] Wolfe JM, Horowitz TS, Van Wert MJ, Kenner NM, Place SS, Kibbi N (2007). Low target prevalence is a stubborn source of errors in visual search tasks. Journal of Experimental Psychology: General.

[CR37] Wolfe JM, Van Wert MJ (2010). Varying target prevalence reveals two dissociable decision criteria in visual search. Current Biology.

